# Flap technique-assisted surgeries for advanced retinitis pigmentosa complicated with macular hole: a case report and literature review

**DOI:** 10.1186/s12886-021-02082-3

**Published:** 2021-09-06

**Authors:** Chia-Ying Lee, Chung-May Yang, Chang-Hao Yang, Fung-Rong Hu, Ta-Ching Chen

**Affiliations:** 1 Department of Medical Education, National Taiwan University Hospital, Taipei, Taiwan; 2 Department of Ophthalmology, National Taiwan University Hospital, Medical College, National Taiwan University, 7 Chung-Shan S. Road, Taipei, Taiwan; 3 Department of Ophthalmology, College of Medicine, National Taiwan University, Taipei, Taiwan

**Keywords:** Retinitis pigmentosa, Macular hole, Lens capsular flap transplantation, Inverted internal limiting membrane flap

## Abstract

**Background:**

Full-thickness macular hole (FTMH) is a rare complication in retinitis pigmentosa (RP) patients and may increase intraoperative challenges. Furthermore, lens capsular flap transplantation and inverted internal limiting membrane (ILM) flap were reported to close complicated FTMH successfully. Here, we present a case of bilateral advanced RP complicated by a FTMH treated with a novel lens capsular flap transplantation and inverted internal limiting membrane flap.

**Case presentation:**

A 46-year-old presented to our hospital with a complaint of progressively blurred vision and metamorphopsia in both eyes. Spectral-domain optical coherence tomography revealed a FTMH with retinoschisis in the right eye and another FTMH in the left eye. ILM peeling with inverted ILM flap technique was performed on the right eye and ILM peeling with anterior lens capsular flap technique was performed on the left eye. Post-operative follow-up showed successful closure of the FTMH and improved vision in both eyes.

**Conclusions:**

In our present case, flap-assisted techniques for retinitis pigmentosa with macular hole result in excellent visual and anatomic outcomes.

**Supplementary Information:**

The online version contains supplementary material available at 10.1186/s12886-021-02082-3.

## Background

Retinitis pigmentosa (RP) is a type of inherited retinal disease with an estimated prevalence of 1 in 4000 [1]. RP causes loss of peripheral vision and night blindness; thus, evaluation and preservation of central vision are critical [1, 2]. Macular abnormalities are not uncommon among RP patients and were found to be more frequent than the general population, with a reported prevalence of 7.4−43.8 % [3–5]. Cystoid macular edema (CME), epiretinal membrane (ERM), and vitreomacular traction (VMT) are the most common macular abnormalities in RP, with a prevalence of 5–20 % [3–8], 0.6−27.3 % [3–5, 8], 0.8−5 % [3, 5, 8] respectively, in the general RP population.

Compared to CME, ERM, and VMT, full-thickness macular hole (FTMH) is a relatively rare complication of RP, with a reported prevalence of 0.5−4.5 % [3–6, 8] and only several small case series have been reported. Surgical treatment for FTMH in patients with RP is more difficult than that in general patients. Dysfunction of the retinal pigment epithelium (RPE) in RP may lead to a failure of the pumping mechanism, causing hydration of the foveolar with progressive enlargement of the hole and leakage of fluid through the RPE [9, 10]. Furthermore, pigmentary changes in the macula area may further increase the challenges intraoperatively.

Recently, an inverted internal limiting membrane (ILM) flap was successfully applied for complicated FTMH with good results [11]. Furthermore, lens capsular flap transplantation was reported to successfully close refractory FTMHs and posterior retinal holes, serving as an alternative when previous ILM flaps fail [12, 13]. Herein, we present a case of RP complicated with bilateral FTMH who underwent surgery using inverted flap and lens capsular flap techniques. In addition, we conducted a comprehensive literature review on the treatment and outcome of past cases of RP with FTMH. To the best of our knowledge, this is the first report regarding flap technique-assisted surgeries for complicated FTMH with RP.

## Case presentation

A 46-year-old male presented to our hospital with a complaint of progressively blurred vision and metamorphopsia in both eyes. He also suffered from night blindness since childhood and had been diagnosed with RP with a positive family history. Otherwise, he denied any history of other systemic diseases.

At presentation, his best corrected visual acuity (BCVA) was 20/400 in the right eye and 20/400 in the left eye (Snellen). Anterior segment examination revealed dense nuclear sclerosis in both eyes. With dilated fundoscopy, both eyes presented advanced RP, including peripheral retinal atrophy with bone spicule-shaped pigmentation in the mid-periphery, waxy pallor of the optic nerve head, and attenuation of retinal vessels. Severe macular involvement with profound pigmentary change was also noted in both eyes. Spectral-domain optical coherence tomography revealed a stage IV FTMH of 320 μm in diameter with retinoschisis in the right eye and another stage IV FTMH of 495 μm in diameter in the left eye. The axial length was 23.24 mm in his right eye and 23.43 mm in his left eye. Electroretinography showed diminished waves, both in rods and cones, in both eyes.

In his right eye with cataract, FTMH, and retinoschisis, 25-gauge pars plana vitrectomy, ERM/ILM peeling with inverted ERM/ILM flap technique, and C_3_F_8_ gas tamponade were performed combined with phacoemulsification and posterior chamber intraocular lens implantation under retrobulbar anesthesia. Indocyanine green (ICG) staining was used intraoperatively for ILM peeling.

In his left eye, 25-gauge pars plana vitrectomy, ERM/ILM peeling with anterior lens capsular flap technique, and C_3_F_8_gas tamponade were performed combined with phacoemulsification and posterior chamber intraocular lens implantation under retrobulbar anesthesia. The technique of lens capsular flap was similar to that presented by Yang in 2016 [12]. In brief, the circular anterior capsular flap was harvested by continuous circular capsulorhexis, and then it was stained with 0.125 % ICG and preserved in a balanced salt solution. Afterward, the flap was cut into a size a little larger than that of the FTMH using scissors and was translocated to the FTMH using micro forceps. Both surgical procedures were demonstrated on video (see Additional file 1).



**Additional file 1.**



**Fig. 1:**
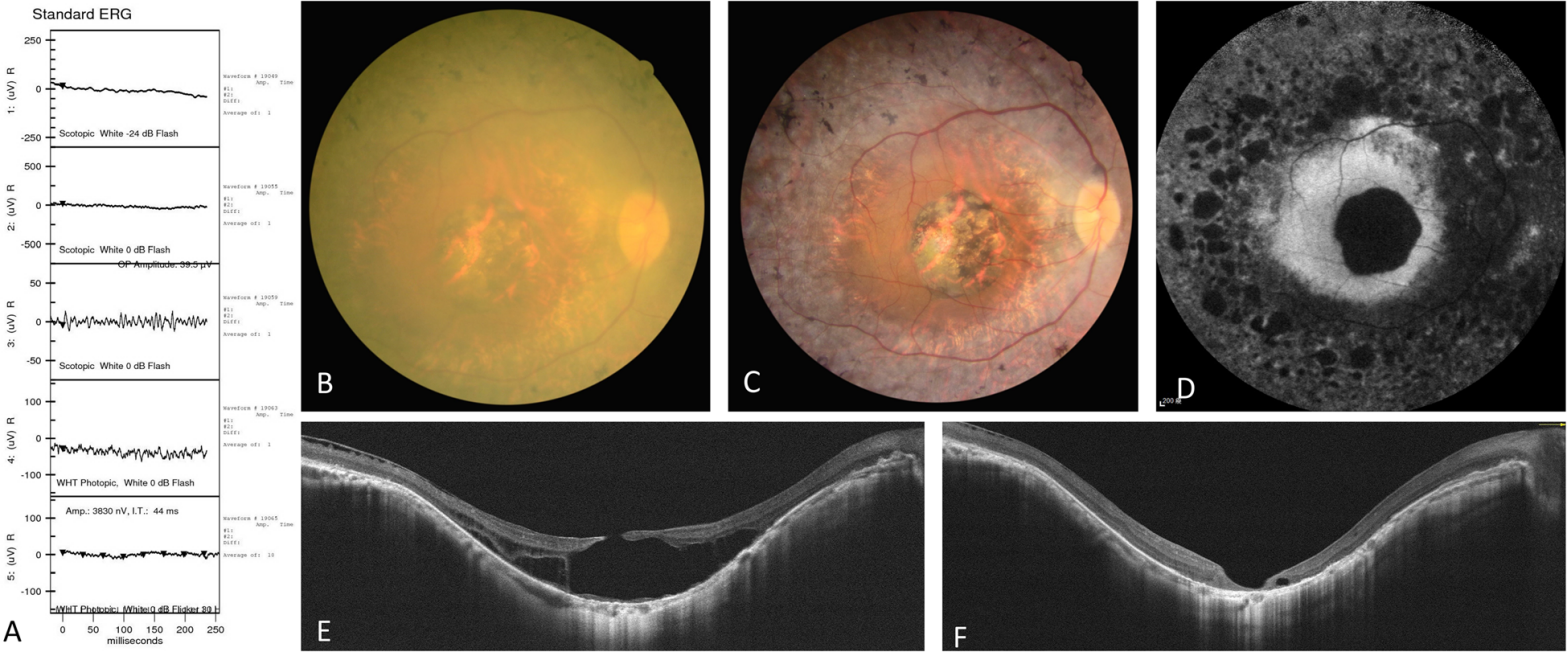
Pre-operative and post-operative changes of the right eye. (**A**) Pre-operative electroretinography. (**B**) Fundus photograph before surgery. (**C**) Fundus photograph 3 months after surgery. (**D**) Fundus autofluorescence after surgery. (**E**) Horizontal optical coherence tomography before surgery. (**F**) Horizontal optical coherence tomography 3 months after surgery

**Fig. 2:**
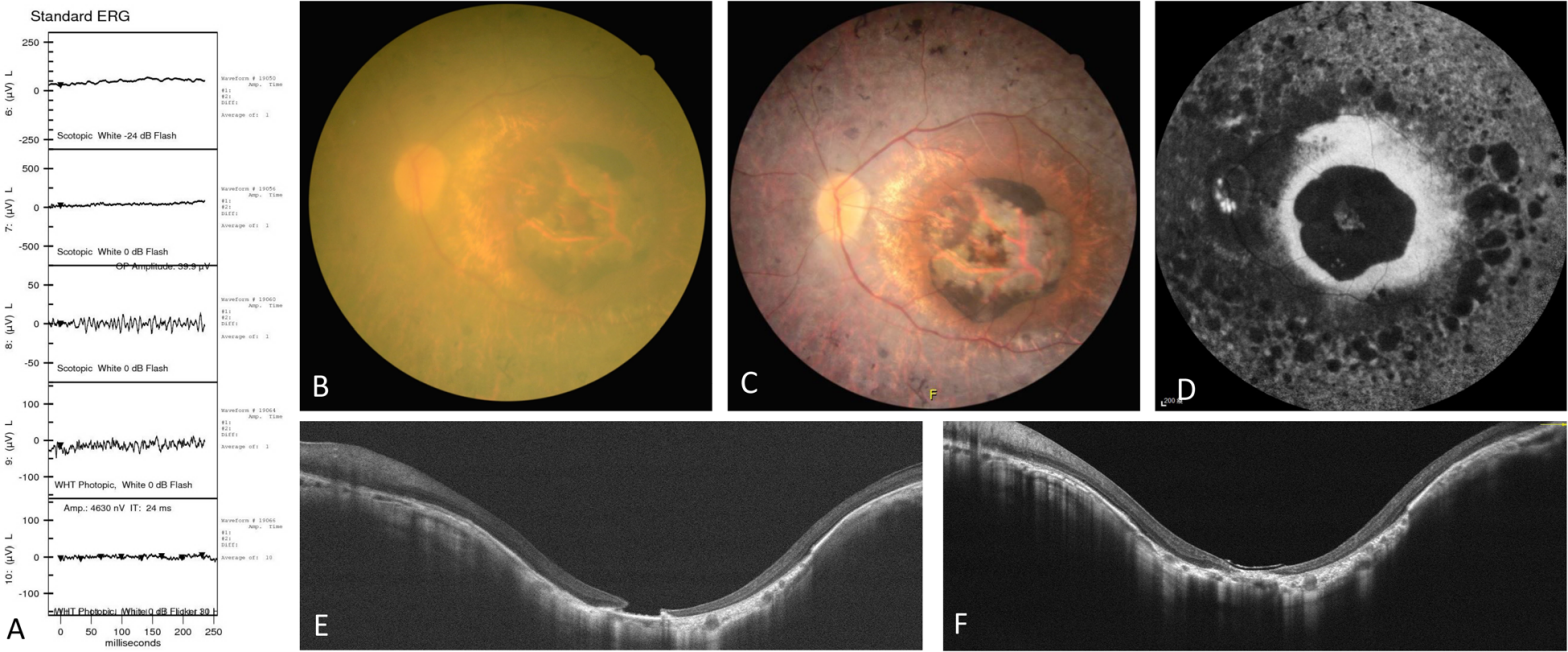
Pre-operative and post-operative changes of the left eye. (**A**) Pre-operative electroretinography. (**B**) Fundus photograph before surgery. (**C**) Fundus photograph 1 month after surgery. (**D**) Fundus autofluorescence after surgery. (**E**) Horizontal optical coherence tomography before surgery. (**F**) Horizontal optical coherence tomography 1 month after surgery

Three months after surgery of the right eye, OCT confirmed successful closure of the FTMH, and BCVA improved to 20/100. One month after surgery of the left eye, OCT confirmed successful closure of the FTMH, and BCVA improved to 20/125. The pre-operative and post-operative imaging are shown in Figs. 1 and 2.

## Discussion and conclusion

FTMH is a rare but sight-threatening condition in RP patients. Among the macular interface abnormalities in patients with RP, CME and ERM are the most common findings, while FTMH is less common, with a prevalence of 0.5−4.5 % [3–6, 8]. Given the relative rarity of FTMH in RP, to date, only small sample-sized studies have reported cases of FTMH in RP, and no large-size cohort study was found in our comprehensive literature search.

According to our literature analysis, ten studies that reported the surgical course and outcome of RP patients with FTMH were identified and analyzed (Table 1). A total of 24 patients and 24 eyes were included in this study. Among the 24 eyes that underwent vitrectomy and ILM peeling, 15 (62.5 %) patients had improved vision, 5 (20.8 %) remained unchanged, and 4 (16.7 %) worsened. Twenty (83.3 %) cases had complete closure of the hole, three (12.5 %) cases failed to close the FTMH, and 1 (4.2 %) reopened 2 years later. Vitreous surgery has been suggested by several studies as a proper treatment for RP patients with FTMH, in which most patients achieved vision improvement and FTMH closure. Patients with complications, such as posterior vitreous detachment, retinal detachment, and CME, did not hinder the success of FTMH closure. It was presumed that failed closure and worsened vision was due to retinal thinning and atrophy at the macula caused by long-term damage to the photoreceptor or RPE loss [5, 14]. In general, FTMH is commonly treated with vitrectomy with or without ILM peeling. Closure rates were reported to reach 90 %; however, failure of hole closure is higher in cases with high myopia [15, 16], chronic FTMH [17] and diabetic retinopathy [18].

**Table 1:** Case review of surgical treatment and postoperative outcome of RP with FTMH

Author	Year	Origin of population	Subject number	Number of eyes	Associated disease	Surgical method	BCVA change	OCT change
Vingolo et al. [19]	2015	Italy	3	3		PPV, ILM peeling, gas tamponade	3 improved	3 complete FTMH closure
Jin et al. [20]	2008	Japan	4	4	PVD, high myopia, RD, CME	PPV, ILM peeling, C3F8 injection	3 improved, 1 improved partially	4 complete FTMH closure
Panagiotou et al. [2]	2020	Greece	1	1	PVD, absence of EZ	PPV, ILM peeling, C2F6 injection	1 unchanged	1 complete FTMH closure
Hagiwara et al. [5]	2011	Japan	2	2	RPE atrophy	PPV, ILM peeling, SF6 injection	1 improved, 1 worsened	2 complete FTMH closure
Liu et al. [21]	2020	China	2	2	LHEP, RD, foveal detachment	1. PPV, ILM peeling, LHEP transplantation, autologous blood, C3F8 injection2. PPV, ILM peeling, laser, LHEP transplantation, C3F8 injection	2 improved	2 complete FTMH closure
Yan et al. [14]	2018	China	4	4		PPV, ILM peeling, SF6 or C3F8 or silicon injection	2 improved, 1 unchanged, 1 worsened	3 complete FTMH closure,1 remained unclosed
Ratra et al. [22]	2013	India	5	5	CME	PPV, ILM peeling, C3F8 or silicon injection	2 improved, 1 unchanged, 2 worsened	3 complete FTMH closure,2 remained unclosed
Enani et al. [23]	2017	Saudi Arabia	1	1		PPV, ILM peeling, gas tamponade	1 unchanged	1 complete FTMH closure
García-Fernández et al. [24]	2013	Spain	1	1	SRF, PVD	PPV, ILM peeling, SF6 injection	1 unchanged	1 reopened 2 years later
Amemiya et al. [25]	2002	Japan	1	1		PPV, ILM peeling, gas tamponade	1 improved	1 complete FTMH closure

*PPV* pars plana vitrectomy; *ILM* internal limiting membrane; *FTMH* full-thickness macular hole; *PVD* posterior vitreous detachment; *RD* retinal detachment; *CME* cystoid macular edema; *EZ* ellipsoid zone; *RPE* retinal pigment epithelium; *LHEP* lamellar-hole associated epiretinal proliferation

In our present case, though the axial length was only 23.24 mm in his right eye and 23.43 mm in his left eye, large posterior staphyloma with significant chorioretinal atrophy was noted in his both eyes. The configuration was quite similar to the high myopia-related FTMH. Surgical repair for this kind of FTMH is challenging because of the poor retinal adhesion to the underlying surface and the posterior staphyloma that may produce an inverse traction to impede retinal adhesion [26–28]. However, to seal the FTMHs is important in order to maintain the remnant central vision and prevent the further degeneration of photoreceptors over the long-term [14, 21]. ILM flap technique was proven to be effective in this circumstance [29–31]. In his right eye, tangential traction exerted by the ERM could be proposed as a causative factor in the development of a macular hole–related retinoschisis. Therefore, inverted flap technique was chosen to make sure the successful closure of FTMH with resolution of retinoschisis. The post-operative vision improved to 20/100 from 20/400 with concomitant removal of cataract.

A diameter of FTMH over 500 μm could be considered a risk factor for unclosure after primary vitrectomy and flap-related techniques can improve the closure rate [32, 33]. However, if ILM was not available such as being peeled in previous surgeries or other causes, lens capsular flap transplantation, which has been first introduced by Yang in 2016, could be a good alternative to overcome the aforementioned difficulties. The lens capsule is thicker and has a higher density than the ILM, enabling it to settle down easier on the retinal surface and be directed to the target place [12, 34]. For his left eye, we chose to use lens flap for his left eye rather than inverted ILM flap in order not to damage the atrophic retina in the circumstance of severely pigmentary change which hindered the visualization of ILM. The post-operative vision improved to 20/125 from 20/400 with concomitant removal of cataract and cell migration was shown beneath the lens flap.

The pathophysiology of FTMH formation in RP remains unclear. Multifactorial pathogenesis is widely proposed in several studies [4]. It is hypothesized that degenerative vitreous, with the collapse of the vitreous gel and posterior vitreous detachment, may lead to vitreomacular interface changes, which is likely to contribute to CME formation. A case reported that Muller cells, glial cells, and fibroblasts were found in the removed membrane, suggesting that the FTMH was caused by vitreous degeneration due to RP [25]. Other pathophysiologic changes in RP, including a breakdown of the blood-retinal barrier, Muller cell dysfunction and swelling, failure of RPE pumping, and antiretinal antibodies, were also presumed to lead to CME formation [35]. As a result, rupture and posterior fusion of the cysts of a CME may facilitate FTMH formation [6, 25, 36–38]. High myopia with globe elongation macular retinoschisis may also predispose the formation of FTMH [21].

In conclusion, FTMH is a rare macular abnormality in patients with RP. Previous studies have shown successful outcomes with pars plana vitrectomy. We propose the first case report that flap-assisted techniques may serve as an alternative for advanced RP with FTMH. More studies are warranted in the future to verify the anatomical effectiveness and functional benefits of flap-assisted techniques for RP with FTMH.

## Data Availability

All data generated or analyzed during this study are included in this published article and its supplementary information files.

## Abbreviations

RP: Retinitis pigmentosa
ILM: Internal limiting membrane
FTMH: Full-thickness macular hole
CME: Cystoid macular edema
ERM: Epiretinal membrane
VMT: Vitreomacular traction
RPE: Retinal pigment epithelium
BCVA: Best corrected visual acuity
ICG: Indocyanine green
